# Effective enhancement of *Pseudomonas stutzeri* D-phenylglycine aminotransferase functional expression in *Pichia pastoris* by co-expressing *Escherichia coli* GroEL-GroES

**DOI:** 10.1186/1475-2859-11-47

**Published:** 2012-04-19

**Authors:** Kanidtha Jariyachawalid, Poramaet Laowanapiban, Vithaya Meevootisom, Suthep Wiyakrutta

**Affiliations:** 1 Department of Biotechnology, Faculty of Science, Mahidol University, Rama 6 Road, Bangkok, 10400, Thailand; 2 Department of Microbiology, Faculty of Science, Mahidol University, Rama 6 Road, Bangkok, 10400, Thailand; 3 Center of Excellent for Agricultural Biotechnology: (AG-BIO/PERDO-CHE), Bangkok, Thailand

## Abstract

**Background:**

D-phenylglycine aminotransferase (D-PhgAT) of *Pseudomonas stutzeri* ST-201 catalyzes the reversible stereo-inverting transamination potentially useful in the application for synthesis of D-phenylglycine and D-4-hydroxyphenylglycine using L-glutamate as a low cost amino donor substrate in one single step. The enzyme is a relatively hydrophobic homodimeric intracellular protein difficult to express in the soluble functionally active form. Over-expression of the *dpgA* gene in *E. coli* resulted in the majority of the D-PhgAT aggregated into insoluble inclusion bodies that failed to be re-natured. Expression in *Pichia pastoris* was explored as an alternative route for high level production of the D-PhgAT.

**Results:**

Intracellular expression of the codon-optimized synthetic *dpgA* gene under the *P*_*AOX1*_ promoter in *P. pastoris* resulted in inactive D-PhgAT associated with insoluble cellular fraction and very low level of D-PhgAT activity in the soluble fraction. Manipulation of culture conditions such as addition of sorbitol to induce intracellular accumulation of osmolytes, addition of benzyl alcohol to induce chaperone expression, or lowering incubation temperature to slow down protein expression and folding rates all failed to increase the active D-PhgAT yield. Co-expression of *E. coli* chaperonins GroEL-GroES with the D-PhgAT dramatically improved the soluble active enzyme production. Increasing gene dosage of both the *dpgA* and those of the chaperones further increased functional D-PhgAT yield up to 14400-fold higher than when the *dpgA* was expressed alone. Optimization of cultivation condition further increased D-PhgAT activity yield from the best co-expressing strain by 1.2-fold.

**Conclusions:**

This is the first report on the use of bacterial chaperones co-expressions to enhance functional intracellular expression of bacterial enzyme in *P. pastoris*. Only two bacterial chaperone genes *groEL* and *groES* were sufficient for dramatic enhancement of functionally active D-PhgAT expression in this yeast. With the optimized gene dosage and chaperone combinations, *P. pastoris* can be attractive for intracellular expression of bacterial proteins since it can grow to a very high cell density which is translated into the higher volumetric product yield than the *E. coli* or other bacterial systems.

## Background

**Figure 1:**
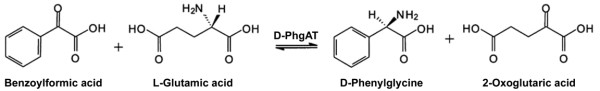
**Stereo-inverting aminotransferase reaction catalyzed by D-PhgAT from *Pseudomanas stutzeri *ST-201.** The enzyme reversibly transfers the amino group between L-glutamic acid and D-phenylglycine which have opposite stereochemistry.

The D-phenylglycine aminotransferase (D-PhgAT) from a soil bacterium *Pseudomonas stutzeri* ST-201 [1], catalyzes the reversible transamination specific for D-phenylglycine or D-4-hydroxyphenylglycine in which 2-oxoglutarate is an amino-group acceptor that are converted into benzoylformate or 4-hydroxybenzoylformate and L-glutamate (Figure 1). Both D-phenylglycine and D-4-hydroxyphenylglycine are important side chain moieties in high demand for production of semisynthetic penicillin and cephalosporin antibiotics such as ampicillin, amoxicillin, cephalexine and cephadroxyl. Currently, D-phenylglycine and D-4-hydroxyphenylglycine are industrially produced by a process using two enzymes, the hydantoinase and carbamoylase [2]. However, the reaction rates are low since the solubilities of the substrates, (D,L) phenylhydantoin and (D,L)-4-hydroxyphenylhydantoin, are very poor. Alternatively, a single-step enzymatic synthesis using a D-amino acid aminotransferase is possible. But known D-amino acid aminotransferases have very low transamination activity towards D-phenylglycine [3] and they accept only D-amino acids as the amino-group donors. Due to the high cost of D-amino acids (such as D-alanine, D-glutamic acid), their direct application as amino-group donors is impractical. Using an L-amino acid (such as L-alanine or L-glutamic acid) together with an amino acid racemase to convert it to the corresponding D-amino acid to serve as the amino-group donor for the D-amino acid aminotransferase will increase the production cost. Additionally, the racemase may interfere with the transamination reaction. By contrast, with the D-PhgAT, due to its characteristic “stereo-inverting” transamination activity, a low cost substrate L-glutamic acid can be used as an amino-group donor for the synthesis of enantiomerically pure D-phenylglycine or D-4-hydroxyphenylglycine in a single transamination step without the need for any amino acid racemase [1].

Before enzymatic synthesis of D-phenylglycine and D-4-hydroxyphenylglycine using D-PhgAT can be further developed, a large supply of the enzyme should be established. Initially, the conventional method using commercially available gene expression systems for over-production of the D-PhgAT in *E. coli* resulted in the formation of inclusion body and very low amount of the soluble functional enzyme was obtained [unpublished observations]. The attempt to solubilize the insoluble enzyme aggregates and refold the protein, using a published method [4], to the functional D-PhgAT was not successful.

It is well-known that *Pichia pastoris* protein expression system offers various advantages [5-7] including the success in high level production of a variety of heterologous proteins both intra- and extracellularly, the ability of the host to grow to very high cell density using simple culture media, and the availability of powerful genetic manipulation techniques. Several approaches for improving the solubility and expression level of recombinant protein in *P. pastoris* have been reported, for instance, low-temperature expression [8,9], adjusting of media composition [10,11], modifying the host strains [12,13], fine-tuned gene expression by generating promoter mutants [14], rational site-directed mutagenesis [15,16], directed evolution [17,18], translational fusion proteins [19-22], co-expression of chaperones [23-26], codon optimization [27,28] and creating the multiple gene copy number [29-31]. The beneficial characteristics and the documented successful applications encouraged us to exploit the *P. pastoris* protein expression system for production of soluble active D-PhgAT at high yield.

## Results

### Intracellular expression of D-PhgAT in *P. pastoris* KM71

**Figure 2:**
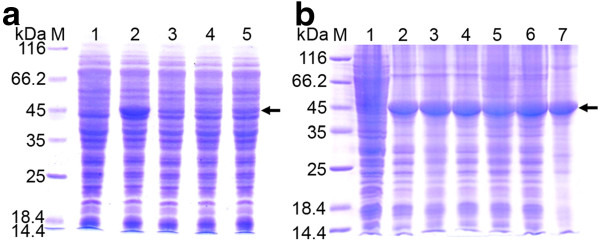
**Expression of D-PhgAT in *P. pastoris *KM71.** SDS-PAGE analysis of total cellular proteins of (**a**) *P. pastoris* KM71 expressing wild-type and codon-optimized *dpgA* genes under the control of *AOX1* promoter at 30°C for 24 h. Lane M, molecular mass markers; Lane 1, *P. pastoris* KM71 harboring the parent vector pPIC3.5 K; Lane 2, *P. pastoris* strain KM_AT3 (see Table 1) expressing D-PhgAT from the codon-optimized *dpgA* gene; Lane 3–5, three *P. pastoris* clones expressing D-PhgAT from the wild-type *dpgA* gene. (**b**) Lane 2–7, *P. pastoris* KM_AT3 after induction at 30°C for 24, 48, 72, 96, 120 and 144 h, respectively. The arrow indicates protein bands of the D-PhgAT (45 kDa).

Levels of D-PhgAT production from *P. pastoris* KM71 clones harboring 3 copies of wild-type and codon-optimized *dpgA* genes under the *AOX1* promoter were compared. Expression from the codon-optimized *dpgA* gene yielded high amount of D-PhgAT protein (Figure 2A) with low level of D-PhgAT activity (Table 1, strain KM_AT3). By contrast, expression from wild-type *dpgA* gene resulted in much lower amount of D-PhgAT protein and no D-PhgAT activity was detectable.

**Table 1:** **Copy number of *dpgA*** and *groEL*-*groES***genes and D-PhgAT activity of different ***P. pastoris*** clones**

**Strain name**	**Host strain**	**Integrated plasmid**	**Integration site**	**Copy number**		**Specific D-PhgAT activity [UÂ·mg**^**-1**^**]**^**a**^	**Volumetric D-PhgAT activity** **[UÂ·L**^**-1**^**]**^**b**^
				***dpgA***	***groEL-groES***		
KM_AT3	KM71	pPIC3.5K_	*HIS4*	3	0	0.0054	2.9
		D-PhgAT					
KM_AT1_ELS1	KM71	pAO_	*HIS4*	1	1	12.6	7531
		D-PhgAT_GAP *_*ELS					
KM_AT3_ELS3	KM71_D-PhgAT	pPICZ_26S_GAP_ELS	5′*AOX1*	3	3	23.3	14558
KM_AT3_ELS4			5′*AOX1*	3	4	45.3	34809
KM_AT3_ELS2			26S rDNA	3	2	14.6	11027
KM_AT3_ELS10			26S rDNA	3	10	60.1	41730

Cells were cultured at 30Â°C.

^a^ Specific activity was calculated as unit of D-PhgAT activity per mg of soluble cellular protein.

^b^ Volumetric activity was calculated as unit of D-PhgAT activity obtained per liter of *P. pastoris* culture.

**Figure 3:**
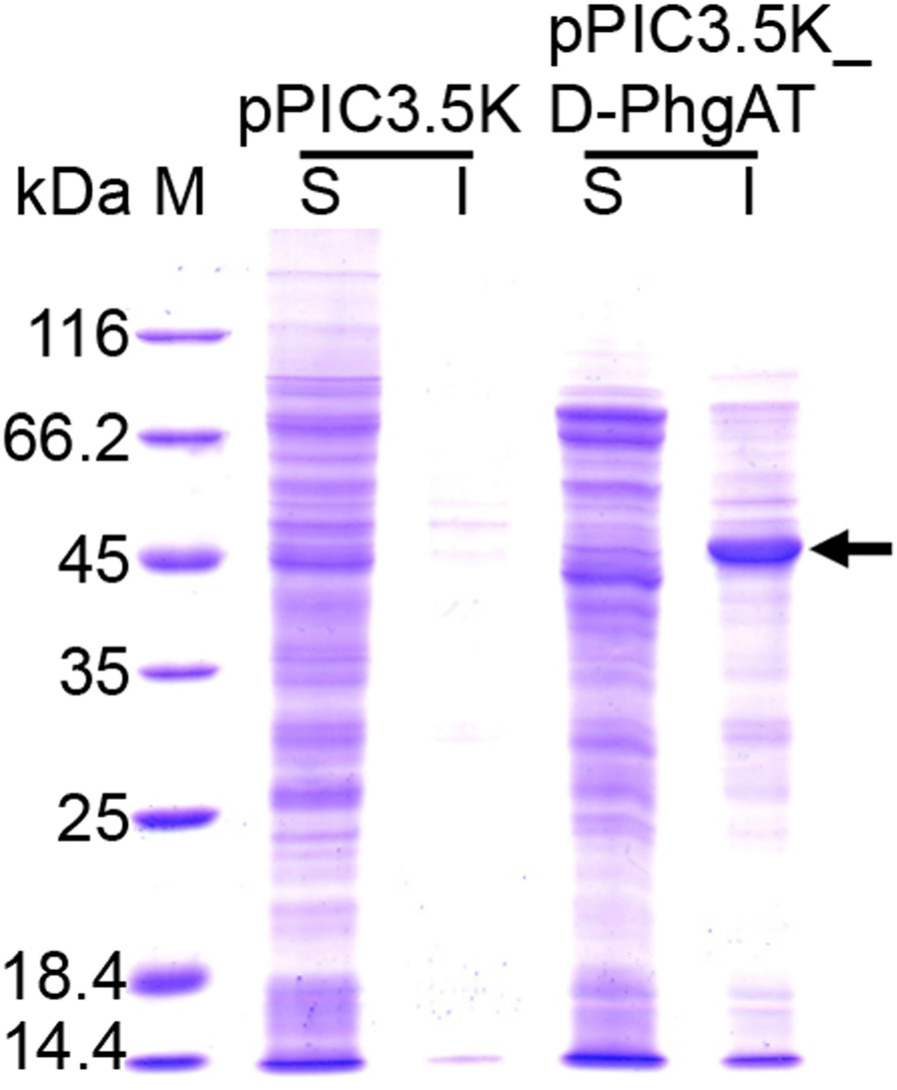
***P. pastoris*KM71 expressed D-PhgAT as insoluble protein.** SDS-PAGE analysis of soluble (S) and insoluble (I) protein fractions of *P. pastoris* KM71 expressing D-PhgAT under the control of *AOX1* promoter after induction at 30°C for 24 h. Lane M, molecular mass markers. The arrow indicates protein band of the D-PhgAT (45 kDa).

**Figure 4:**
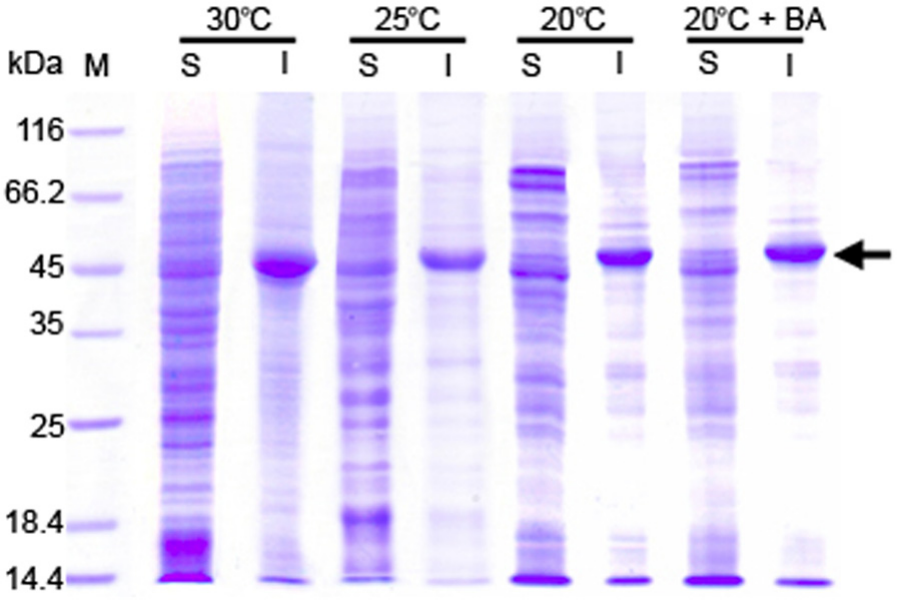
**Effect of temperature and benzyl alcohol (BA) on soluble D-PhgAT production.** SDS-PAGE analysis of soluble (**S**) and insoluble (**I**) protein fractions from *P. pastoris* KM71 expressing D-PhgAT under the control of *AOX1* promoter after induction for 24 h at 30°C, 25°C, and at 20°C without and with 0.4 mM benzyl alcohol added to the culture. Lane M, molecular mass markers. The arrow indicates protein bands of D-PhgAT.

The codon-optimized synthetic *dpgA* gene under the control of the *AOX1* promoter of the pPIC3.5 K plasmid was integrated into the *HIS4* locus on the chromosome of the *P. pastoris* KM71 host. The clone harboring 3 copies of the *dpgA* gene was induced with 0.5% methanol for gene expression at 30°C for 144 h to determine the D-PhgAT yield and the optimal time for product harvesting. The D-PhgAT was strongly expressed constituting up to 30% of total cellular proteins after 24 h of induction and remained at this level until 144 h (Figure 2B). However, majority of the expressed D-PhgAT associated with the insoluble cellular fraction (Figure 3) as inactive enzyme. Soluble protein fraction was found to contain D-PhgAT enzymatic activity at 0.0054 U·mg^-1^ (Table 1). Several strategies known to promote native folding of the over-expressed proteins including lower temperature, increased intracellular concentrations of osmolytes and molecular chaperones [32] were attempted in this study. By using 0.5 M sorbitol, 0.4 to 4 mM benzyl alcohol, and 20°C incubation temperature; the yield of soluble active D-PhgAT was not significantly enhanced as judged from the intensity of protein bands (Figure 4).

### Co-expression of GroEL-GroES chaperones with D-PhgAT in *P. pastoris* KM71

**Figure 5:**
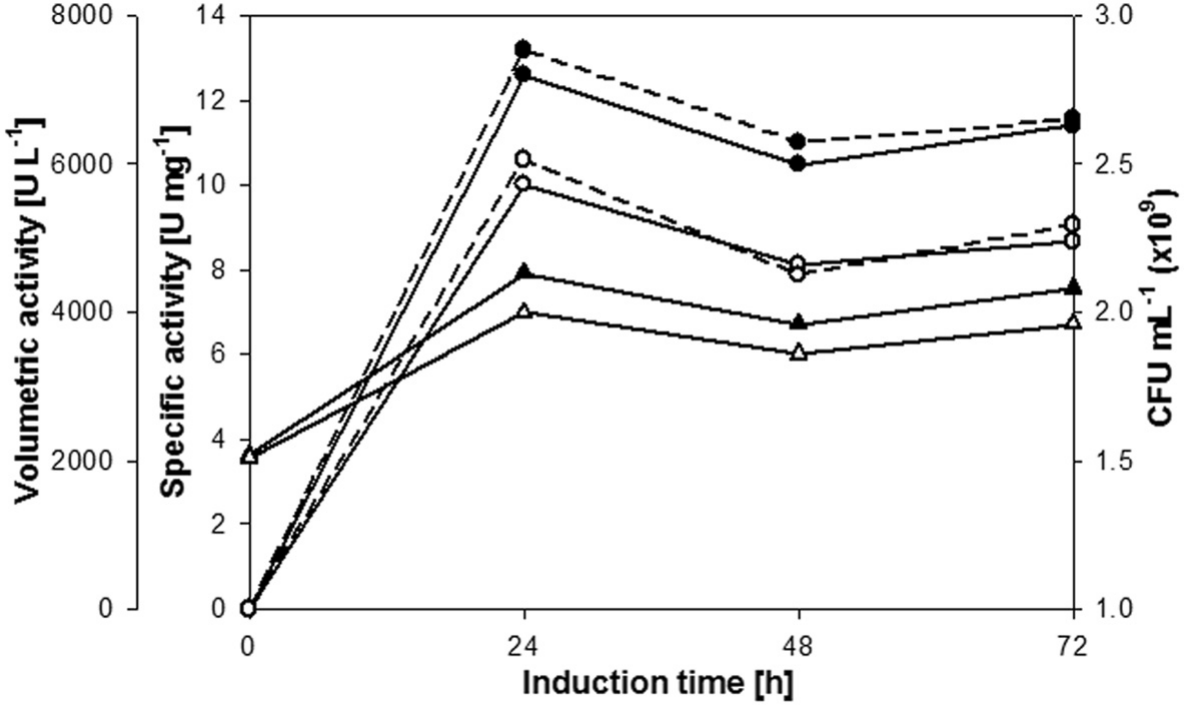
**D-PhgAT activity from *P. pastoris* KM71 co-expressing *groEL *and *groES *with *dpgA.*** D-PhgAT activity in cell extract and viable cell counts of *P. pastoris* KM71 integrated with pAO_D-PhgAT_AOX_ELS plasmid (white circle, white triangle) or pAO_D-PhgAT_GAP_ELS (filled circle, filled triangle) during induction with 0.5% methanol at 30°C for 72 h. Solid line, specific activity; dash line, volumetric activity; please see Table 1 for definitions.

**Figure 6:**
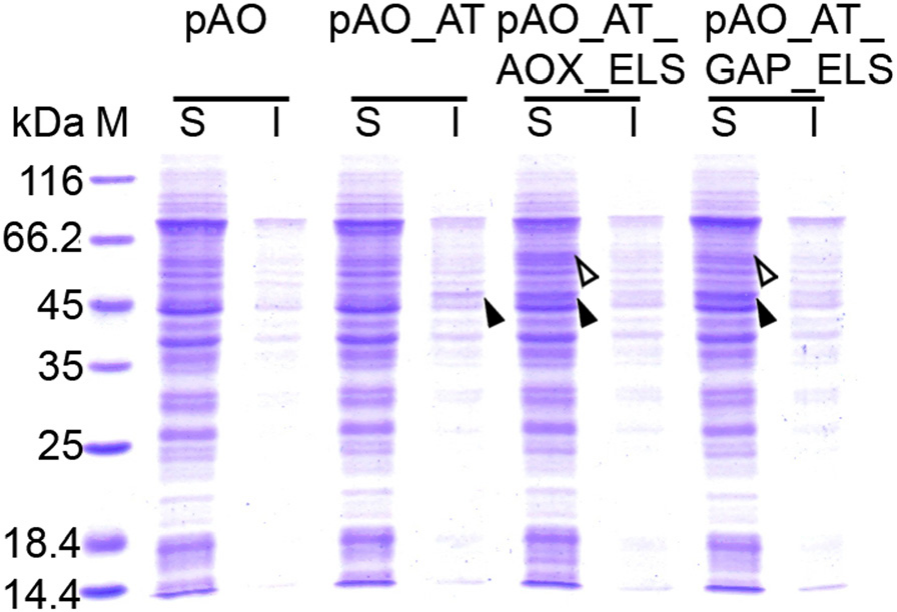
**SDS-PAGE analysis of soluble (S) and insoluble (I) protein fractions from *P. pastoris *KM71 expressing D-PhgAT alone and with GroEL-GroES co-expression.** Lane M, molecular mass markers; pAO, clone with blank pAO815; pAO_AT, clone expressing *dpgA* under *P*_*AOX1*_; pAO_AT_AOX_ELS, clone expressing *P*_*AOX1 *_*dpgA* with co-expressing *P*_*AOX1*_*groEL* and *P*_*AOX1*_* groES*; pAO_AT_GAP_ELS, clone expressing *P*_*AOX1*_* dpgA* with co-expressing *P*_*GAP*_* groEL* and *P*_*GAP*_* groES*. The protein bands corresponding to D-PhgAT (45 kDa) and GroEL (60 kDa) are arrow indicated.

The pAO815 plasmid containing the *dpgA* gene under the control of *AOX1* promoter and the *groEL* and *groES* genes each under the control of an *AOX1* promoter (pAO_D-PhgAT_AOX_ELS) or a *GAP* promoter (pAO_D-PhgAT_GAP_ELS) were constructed [see Additional file 1]. After transformation and gene integration into the *P. pastoris* KM71 chromosome, it was found that co-expression of GroEL-GroES molecular chaperones with D-PhgAT at 30°C yielded functionally active enzyme with the highest activity at 24 h after induction. Clone harboring pAO_D-PhgAT_GAP_ELS yielded slightly higher D-PhgAT activity than that with pAO_D-PhgAT_AOX_ELS (Figure 5). Noticeably, D-PhgAT activity declined after 48 h and slightly increased after 72 h. Viable cell count showed the decrease in number of viable cells at 48 h and increase at 72 h correlated well with the D-PhgAT activity profile. SDS-PAGE analysis of cellular proteins revealed substantial amounts of D-PhgAT protein in the soluble protein fractions concomitant with its absence from the insoluble fractions (Figure 6).

### Expression of multiple copies of *dpgA, groEL* and *groES* genes in *P. pastoris* KM71

**Figure 7:**
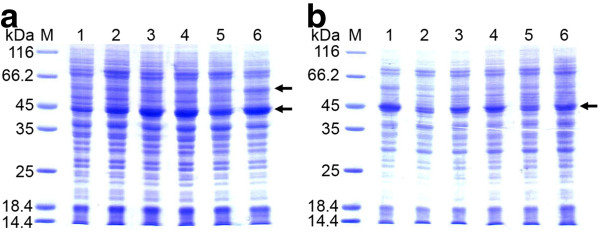
**Comparison of D-PhgAT expressions from *P. pastoris* KM71 strains carrying different copy numbers of* dpgA* and* groEL-groES* genes.** SDS-PAGE analysis of soluble (**a**) and insoluble (**b**) fractions from 6 recombinant strains. Lane M, molecular mass markers; Lane 1, strain KM_AT3; Lane 2; strain KM_AT1_ELS1; Lane 3–6; strains KM_AT3_ELS3, KM_AT3_ELS4, KM_AT3_ELS2, KM_AT3_ELS10, respectively. Please see Table 1 for details of each strain. The protein bands corresponding to D-PhgAT (45 kDa) and GroEL (60 kDa) are arrow indicated.

The *P. pastoris* KM71 chromosomally harboring at the *HIS4* locus 3 copies of the *dpgA* gene under the control of *P*_*AOX1*_ after transformation with pPIC3.5K_D-PhgAT plasmid was used as the host for co-expression with multiple copies of the *groEL* and *groES* genes. The pPICZ_26S rDNA_GAP_GroELS [see Additional file 2] was constructed to express the *P*_*GAP*_*groEL* and *P*_*GAP*_*groES* that could be integrated in multiple copies at either the 5^′^*AOX1* or the 26S rDNA sites of the *Pichia* chromosome. After screening the transformants on YPD plates containing zeocin, two clones from 5^′^*AOX1* integration and two clones from 26S rDNA integration experiments showed resistance to 5000 μg·mL^-1^ of zeocin. Quantitative real time PCR analysis of gene copy number revealed that clones obtained from 5^′^*AOX1* integration contained 3 and 4 copies, while clones from 26S rDNA integration contained 2 and 10 copies of the *groEL*-*groES* genes, repectively. D-PhgAT expressions of these clones were performed at 30°C in culture medium containing 0.5% methanol for 24 h. Compared with strain containing single copies of the *dpgA* and *groEL*-*groES* genes (KM_AT1_ELS1), clones with multiple copies yielded approximately 1.5 times (KM_AT3_ELS2) to 5.5 times (KM_AT3_ELS10) more of D-PhgAT volumetric activity. The D-PhgAT volumetric activity from clone co-expressing 10 copies of *groEL*-*groES* was 14400 times higher than the clone expressing *dpgA* alone (Table 1). SDS-PAGE analyses comparing D-PhgAT expressions from different *P. pastoris* KM71 strains constructed in the present study are shown in Figure 7. The amounts of D-PhgAT protein in the soluble fractions of cellular protein extracts correlated well with the D-PhgAT enzymatic activity summarized in Table 1. And less amounts of insoluble D-PhgAT protein were formed as the copy number of the co-expressing *groEL*-*groES* genes increased.

### Effect of cultivation conditions on soluble active D-PhgAT yield in *P. pastoris* KM_AT3_ELS10

**Figure 8:**
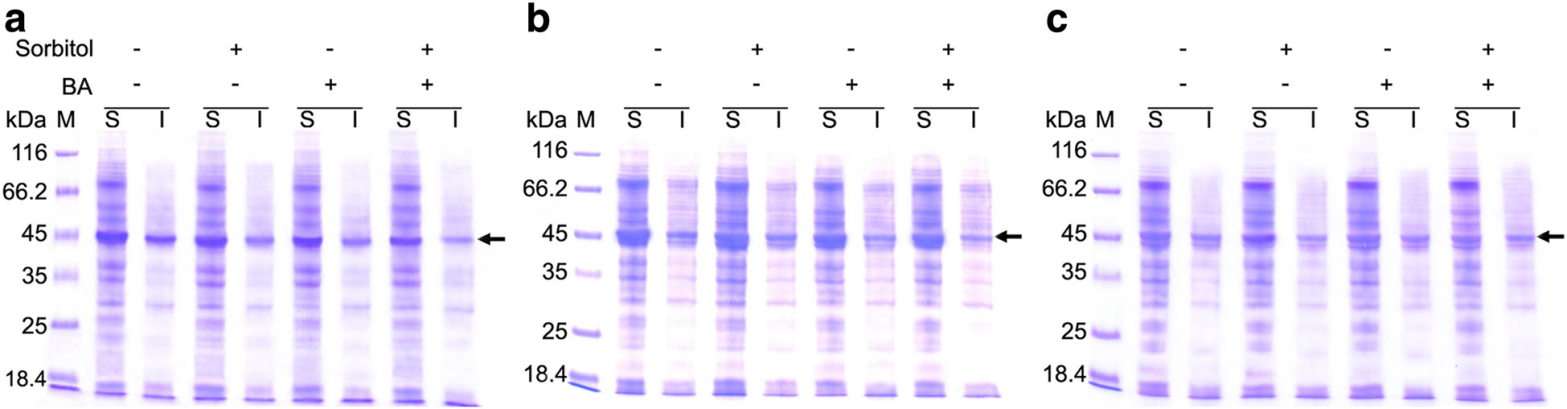
**Comparison of D-PhgAT expressions from * P. pastoris* KM_AT3_ELS10 cultivated under different conditions for 24 h.** SDS-PAGE analysis of soluble and insoluble protein fractions of the cultures induced at 30°C (**a**), 25°C (**b**) and 20°C (**c**), respectively, without and with 0.5 M sorbitol or 0.4 mM benzyl alcohol or both added. Lane M, molecular mass markers; the protein bands corresponding to D-PhgAT (45 kDa) are arrow indicated.

As the highest yield of soluble active D-PhgAT was obtained in the *P. pastoris* KM_AT3_ELS10 which contained 3 copies of the *dpgA* gene and 10 copies of the *groEL-groES* genes, optimization of the cultivation conditions was done on this final co-expressing strain to see if the active D-PhgAT yield could be further increased. The KM_AT3_ELS10 was cultivated under various conditions known to promote the native protein folding including addition of 0.5 M sorbitol, 0.4 mM benzyl alcohol and lowering the incubation temperatures to 25°C and 20°C. At 24 h of induction with 0.5% methanol, cells were collected and the levels of D-PhgAT protein and enzymatic activity were analyzed. As shown in Table 2, lowering the cultivation temperature from 30°C to 25°C resulted in higher D-PhgAT activity yields but further decreasing the temperature to 20°C deteriorated the activity yields to lower than those at 30°C. Addition of 0.5 M sorbitol or 0.4 mM benzyl alcohol or both, impaired the D-PhgAT activity yields at all three temperatures tested. The strongest negative effects were observed when both additives were present. The best specific and volumetric D-PhgAT activity yields of 72.7 U·mg^-1^ and 50512 U·L^-1^, respectively, were obtained when the cultivation temperature was 25°C and without additives. SDS-PAGE analysis of proteins in the soluble and insoluble protein fractions of cells cultivated under the above conditions showed the amount of D-PhgAT protein (Figure 8) in good agreement with the D-PhgAT activity detected (Table 2).

**Table 2:** **Effects of cultivation conditions on D-PhgAT yield from ***P. pastoris*** strain KM_AT3_ELS10**

**Cultivation condition**			**D-PhgAT activity**		
**Temperature**	**Additive**		**Specific activity [U·mg**^**-1**^**]**^**a**^	**Volumetric activity [U·L**^**-1**^**]**^**b**^	**Volumetric productivity[U·L**^**-1**^**·h**^**-1**^**]**
	**0.5 M Sorbitol**	**0.4 mM Benzyl alcohol**			
30°C	**-**	**-**	60.1	41730	1738.75
	+	-	52.3	36526	1521.92
	-	+	52.7	36616	1525.67
	+	+	51.1	35504	1479.33
25°C	-	-	72.7	50512	2104.67
	+	-	70.1	48663	2027.63
	-	+	72.0	50165	2090.21
	+	+	67.1	46602	1941.75
20°C	-	-	58.9	40810	1700.42
	+	-	53.1	36920	1538.33
	-	+	58.3	40830	1701.25
	+	+	51.7	35722	1488.42

^a,b^See Table 1.

### Purification of recombinant D-PhgAT using immobilized metal ion affinity chromatography

**Figure 9:**
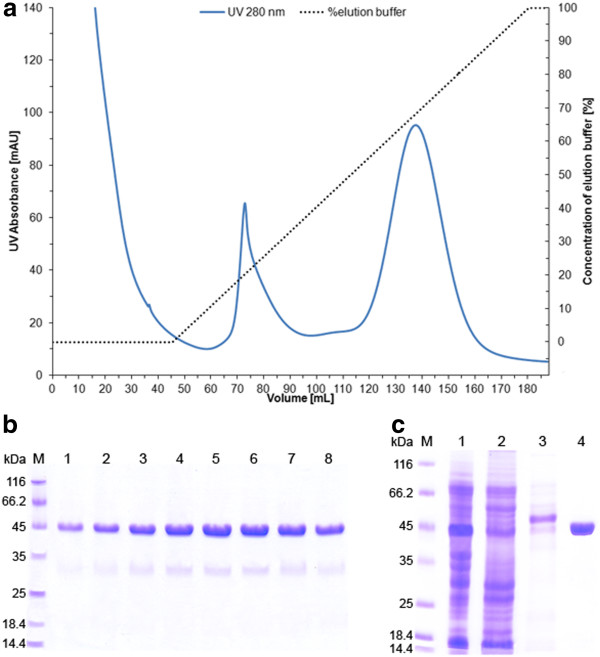
**Purification of the C-terminally 6xHis-tagged D-PhgAT by affinity chromotagraphy.** (**a**) Protein elution profile of D-PhgAT purification from cell-free extract by immobilized ion affinity chromatography; (**b**) SDS-PAGE analysis of eight 3 mL active fractions collected during the elution volume of 130–154 mL showing purity and amount of D-PhgAT (45 kDa) obtained; (**c**) SDS-PAGE analysis of proteins in cell-free extract (Lane 1), unbound fraction (Lane 2), weakly bound fraction (Lane 3), and the purified concentrated D-PhgAT (Lane 4). Lane M, molecular mass markers.

The D-PhgAT was purified from the clarified cell lysates by His tag-mediated affinity chromatography. Protein elution profile during purification is shown in Figure 9A. The unbound proteins were washed out with 45 mL of binding buffer and peak of unrelated proteins was eluted with early imidazole gradient. The D-PhgAT was eluted in a large symmetrical peak well-separated from other host proteins at around 35 mM imidazole. SDS-PAGE analysis of active fractions collected from this peak showed that the D-PhgAT was purified to near homogeneity (Figure 9B). The process brought about 5.2-fold purification and 76.6% yield in one single step. After concentration, the enzyme solution consisting of pure D-PhgAT (Figure 9C) had a specific activity of 888.6 U·mg^-1^.

## Discussion

The methylotrophic yeast *Pichia pastoris* has been developed for heterologous protein expression offering several advantages over other eukaryotic or prokaryotic systems including the high levels expression of intracellular and secreted proteins in the range of grams per liter using the strong inducible *P*_*AOX*_ promoter or the strong constitutive *P*_*GAP*_ promoter, the ability to grow at high cell-density, the simple scale up and well-developed fermentation processes [8,33-35]. Since *P. pastoris* has eukaryotic posttranslational modification machinery which can process the expressed protein similar to higher eukaryotes, it is usually a system of choice for expression of eukaryotic proteins which require proper posttranslational modifications for their functions. Thus, so far there are only a few reports that exploit the benefits of the *P. pastoris* system for the high level expression of bacterial proteins, especially the intracellular ones.

In our case, the *Pseudomonas* D-PhgAT which is a relatively hydrophobic homodimeric intracellular enzyme [1] has been shown to be difficult to express in the regular *E. coli* expression system. We thus explored the possibility of using the *P. pastoris* system as an alternative approach for expressing this problematic enzyme. Initially, the *dpgA* gene encoding the D-PhgAT was expressed under the strong inducible promoter *AOX1* both intracellularly, and extracellularly by fusion with the alpha-factor signal peptide. The extracellular expression resulted in no D-PhgAT protein or enzymatic activity, neither in culture medium nor in cell extract (data not shown). For intracellular expression, the D-PhgAT was highly produced at up to 30% of total cellular proteins but as the inactive enzyme in the insoluble cellular fraction. Noticeably, very low level of D-PhgAT activity could be detected in the cell extracts indicating that functional expression of D-PhgAT in this host is possible which encouraged us to further pursue system optimization. Addition of sorbitol to the culture medium at a high concentration (0.5 M) to force the *Pichia* cells to accumulate viscous intracellular osmolytes (such as glycerol) which slow down the rates of protein structure acquisition and might enhance native folding of the enzyme [32,36,37] yielded no detectable D-PhgAT activity. The reason for this failure may be because intracellular glycerol accumulation in response to osmotic stress might repress the *AOX1* promoter in spite of the presence of methanol as an inducer [38-40]. Likewise, addition of benzyl alcohol at 0.4 – 4 mM as a membrane fluidizing heat shock response inducing agent for inducing the synthesis of intracellular molecular chaperones that would assist protein folding [32,41] was found to be ineffective and all the expressed D-PhgAT was nonfunctional and associated with insoluble cellular fraction. Decreasing the incubation temperature to 20°C to slow down the rates of protein translation and folding could not increase the formation of soluble active D-PhgAT.

As all the simple approaches mentioned above failed, we attempted a more sophisticated method by co-expression with molecular chaperones. D-PhgAT is an enzyme of a Gram-negative bacterium. We hypothesized that if we co-expressed functional *E. coli* chaperones (whose expression plasmids are commercially available) in *P. pastoris* the chaperones would assist native folding of the D-PhgAT. Without codon optimization, two genes encoding the *E. coli* GroEL-GroES chaperonin system were co-expressed intracellularly under the strong inducible *P*_*AOX1*_ or the strong constitutive *P*_*GAP*_ with the *P*_*AOX1*_*dpgA*, each as a single copy gene, in *P. pastoris*. Interestingly, substantial amount of soluble D-PhgAT was produced and ca 2600-fold increased in volumetric activity could be measured, compared with the system without chaperone co-expression (Figures 4, 6 and Table 1). Co-expression of *groEL* and *groES* under the constitutive promoter *P*_*GAP*_ yielded slightly more active D-PhgAT than with the inducible promoter *P*_*AOX1*_ suggesting that constitutive expression of molecular chaperones prior to the strong induction of D-PhgAT expression could be more effective in promoting native protein folding. Increasing gene dosage of the *dpgA* and those of the *groEL* and *groES* progressively increased the D-PhgAT activity yield (Table 1). It was obvious that chaperones gene were required in a higher copy numbers than the *dpgA* gene. The highest gene dosage that could be integrated into the chromosome of the *P. pastoris* host in the present study was 3 copies of the *dpgA* and 10 copies each of the *groEL* and *groES* which could increase the D-PhgAT yield up to 14400-fold higher than with 3 copies of the *dpgA* expressed without chaperone co-expression. It is likely that gene dosage for maximum D-PhgAT yield for this system had not been reached and yet remains to be determined. Co-expression of additional chaperone(s) in various combinations should also be investigated.

## Conclusions

To the best of our knowledge, this is the first report on the use of bacterial chaperone (specifically the *E. coli* GroEL-GroES) co-expression to enhance functional expression of a difficult-to-express bacterial enzyme in *P. pastoris*. The interesting point is that only two bacterial chaperone genes were sufficient to dramatically enhance functional expression of a bacterial enzyme in this host. The *P. pastoris* system is attractive for intracellular expression of bacterial proteins because the organism can be cultured in a very high cell density. With the optimized gene expression this could be translated into a higher volumetric product yield than the bacterial systems such as *E. coli*. Additionally, compared with plasmid-based expression in bacteria, chromosomally integrated recombinant gene in *P. pastoris* can be far more stably expressed in continuous and large-scale fermentation process without the need for antibiotic selection [6].

## Methods

### Strains, plasmids, oligonucleotides, media, chemicals

The *E. coli* DH5α and *P. pastoris* KM71 were obtained from Novagen (Madison, WI, USA) and Invitrogen (San Diego, CA, USA), respectively. The *Pichia* expression plasmids pPIC3.5 K, pAO815, pGAPZ B and pPICZ B were purchased from Invitrogen. The pGro7 was purchased from Takara Bio Inc (Japan). The pPICZ_26S rDNA was constructed and provided by Prof. W. Panbangred (Mahidol University, Thailand). The pBPL-ph plasmid harboring the wild-type *dpgA* gene was obtained from our previous study [42]. The *dpgA* gene was codon-optimized for expression in *Pichia* host, and synthesized by GenScript (Piscataway, NJ, USA) [see Additional file 3. Primers for gene amplification and real-time PCR are shown in Table 3. Restriction endonucleases, DNA polymerase and T4 DNA ligase were supplied by New England Biolabs (Ipswich, MA, USA). Culture media for bacteria and yeast cultivation: Luria-Bertani (LB), Yeast Extract-Peptone-Dextrose (YPD), buffered complex glycerol (BMGY), buffered complex methanol (BMMY) and Minimal Dextrose (MD) were from Becton, Dickinson & Co. (NJ, USA). The antibiotics ampicillin and zeocin were from Bio Basic (Ontario, Canada) and Invitrogen, respectively. Methanol and all other chemicals were obtained from Sigma (St. Louis, MO, USA).

**Table 3:** Primers used in PCR and DNA sequencing reactions. F: forward primer, R: reverse primer, underlined: restriction endonuclease site

**Name**	**Sequence (5**^**′**^**-3**^**′**^**)**	**Application**
Fwt*dpgA*	CGGGATCCACCATGTCGATCCTTAACG	Amplifying and cloning
Rwt*dpgA*	AGGAATTCTCATGATTGGTTTCCAGAC	
F*dpgA*_3.5 K	CGGGATCCACCATGTCCATCCTGAACG	
R*dpgA*_3.5 K	AGGAATTCTTAGCAGTGATGGTGAT	
F*dpgA*_AO	GGAATTCACCATGTCCATCCTGAACGACTACAAGAG	
R*dpgA*_AO	CAGGTCTCCAATTGTTAGCAGTGATGGTGATGGTGATG	
F*groES*_GAP	GGAATTCACCATGAATATTCGTCCATTGC	
R*groES*_GAP	GCTCTAGATTACGCTTCAACAATTGCCAG	
F*groEL*_GAP	GGAATTCACCATGGCAGCTAAAGACGTAAAATTCGGTAACG	
R*groEL*_GAP	GCTCTAGATTACATCATGCCGCCCATGCCACC	
F*groES*_AO	GGAATTCACCATGAATATTCGTCCATTGC	
R*groES*_AO	CAGGTCTCCAATTGTTACGCTTCAACAATTGCCAG	
F*groEL*_AO	GGAATTCACCATGGCAGCTAAAGACGTAAAATTCGGTAACG	
R*groEL*_AO	CAGGTCTCCAATTGTTACATCATGCCGCCCATGCCACC	
F_ELS_PICZ	CCGCTCGAGTTTTGGTCATGCATGAGATC	
R_ELS_PICZ	ATAGTTTAGCGGCCGCTTTTGAAGCTATGGTGTG	
F_GroEL	TGTCCGTACCATGCTCTGAC	Copy number determination
R_GroEL	CAGGTAGCCACGGTCGAAC	
F_D-PhgAT	GCCTGCTCCAGGTGTCTTGC	
R_D-PhgAT	ACTGTCTAGCCAATTCAGCACC	
F_Actin	AAAAGATCTGGCACCACACC	
R_Actin	AGTGGTTCTACCGGAAGCG	
5^′^*AOX1*	GACTGGTTCCAATTGACAAGC	DNA sequencing
3^′^*AOX1*	GCAAATGGCATTCTGACATCC	

### Generation of *Pichia pastoris* strain expressing intracellularly recombinant D-PhgAT

Wild-type *dpgA* gene was amplified from the pBPL-ph plasmid using the *Pfu* DNA polymerase with the Fwt*dpgA* and Rwt*dpgA* primers containing *Bam*HI and *Eco*RI sites, respectively. Cloning of the wild-type *dpgA* gene into the *Pichia* expression plasmid pPIC3.5K, transformation into *P. pastoris* KM71, and selection of integrant clones were performed in the same manners as for the *P. pastoris* codon-optimized synthetic *dpgA* gene described below.

The synthetic *dpgA* gene in the pUC57_D-PhgAT plasmid obtained from GenScript was PCR amplified using the *Pfu* DNA polymerase with the forward and reverse primers F*dpgA*_3.5 K and R*dpgA*_3.5 K (Table 3) having *Bam*HI and *Eco*RI sites, respectively. The PCR product was digested and cloned into the corresponding sites of the *Pichia* expression vector pPIC3.5 K yielding the pPIC3.5K_D-PhgAT which was subsequently transformed into *E. coli* DH5α. The pPIC3.5K_D-PhgAT plasmid extracted from *E. coli* DH5α was linearized with *Sal*I and 10 μg of the DNA was mixed with 80 μL of competent *P. pastoris* KM71 cell suspension. Transformation by electroporation was carried out at 1.5 kV, 25 μF and 200 Ω (Gene Pulser II (Bio-Rad). The transformants were selected on minimal dextrose (MD) without histidine (1.34% YNB with ammonium sulphate without amino acids, 4 x 10^-5^% biotin, and 2% dextrose) agar plates. Two mL of sterile water was spread over the His^+^ transformants on each MD plate to resuspend the transformants. Cell suspensions from all plates were pooled and transferred into a sterile, 50 mL conical centrifuge tube, and vortexed briefly for 5–10 seconds. The cell density was determined using a spectrophotometer (1 OD_600nm_ = 5 x 10^7^ cells·mL^-1^). The 10^-5^, 10^-6^ and 10^-7^ dilutions of the pooled transformants were prepared using sterile water and 200 μL aliquots were plated on YPD agar containing Geneticin at final concentration of 250, 500, 750 and 1000 μg·mL^-1^. All plates were incubated at 30°C and examined daily to select for the strains containing multiple copies of the *dpgA* gene. The integration of the *dpgA* gene into the chromosome of selected *Pichia* transformants was verified by PCR using gene specific primers (F*dpgA*_3.5 K and R*dpgA*_3.5 K, Table 3). The verified clone with the highest D-PhgAT expression level as judged from the enzyme activity was subjected to DNA sequencing to check for the correctness of the integrated *dpgA* gene before it was utilized in this study. Genomic DNA was isolated from the selected *P. pastoris* clones. The integrated DNA fragment containing the *dpgA* gene was amplified using the 5^′^*AOX1* and 3^′^*AOX1* primers. The PCR product was subjected to DNA sequencing on both strands using the 5^′^*AOX1* and 3^′^*AOX1* as sequencing primers (Table 3).

### Construction of plasmids for co-expressing D-PhgAT with GroEL and GroES

The *dpgA* gene amplified from the pUC57_D-PhgAT plasmid was cloned into *Eco*RI site of the pAO815 to construct the pAO_D-PhgAT plasmid. The *groEL* and *groES* genes encoding the GroEL and GroES chaperonins were individually amplified from the pGro7 plasmid using the forward and reverse primers (F*groEL*_GAP, R*groEL*_GAP and F*groES*_GAP, R*groES*_GAP) containing *Eco*RI and *Xba*I sites for cloning separately into pGAPZ B to yield the pGAPZ B_GroEL and pGAPZ B_GroES, respectively. The pGAPZ B_GroES plasmid was cut with *Bgl*II and *Bam*HI and the resulting 1.2 kb of GroES expression cassette was ligated into the *Bam*HI-digested pGAPZ B_GroEL to generate the pGAPZ B_GroEL_GroES. The combined expression cassette of GroEL and GroES was cloned into the *Bam*HI-digested pAO815_D-PhgAT to create the pAO_D-PhgAT_GAP_ELS [see Additional file 1]. The construction of pAO_D-PhgAT_AOX_ELS was done by a similar procedure except that the *groEL* and *groES* genes were cloned into the pAO815, instead of the pGAPZ B, to place the chaperonin genes under the control of the *AOX1* promoter. (In the cloning of *dpgA*, *groEL*, *groES* genes into the pAO815, the pAO815 plasmid was cut with *Eco*RI. The *dpgA*, *groEL*, *groES* were amplified with primers (Table 3) that incorporated an *Eco*RI site at one end and a *Bsa*I site at the other end of the PCR products. The *Bsa*I digested end is compatible with the *Eco*RI overhang and can be ligated to the *Eco*RI site but after ligation the *Eco*RI recognition sequence was destroyed. Orientation of the inserted gene can be determined by double digestions of the recombinant plasmid with *Eco*RI and *Bam*HI.) The pAO_D-PhgAT_GAP_ELS and pAO_D-PhgAT_AOX_ELS plasmids were each linearized with *Stu*I and transformed into *P. pastoris* KM71. Transformants were selected on minimal dextrose (MD) without histidine plates. PCR analyses using gene-specific primers were done to confirm the presence of *dpgA*, *groEL* and *groES* genes integrated in the *P. pastoris* chromosome.

### *In vivo* generation of *P. pastoris* KM71 containing multiple copies of *dpgA*, *groEL* and *groES* genes

The GroEL and GroES expression cassettes were PCR amplified from the pGAPZ B_GroEL_GroES using the forward and reverse primers F_ELS_PICZ and R_ELS_PICZ (Table 3) containing *Xho*I and *Not*I sites, respectively, for cloning into pPICZ_26S rDNA. The resulting pPICZ_26S rDNA_GAP_GroELS [see Additional file 2] was linearized with either *Sac*I or *Sfo*I for integration at the 5^′^AOX1 or 26S rDNA locus, respectively, in the chromosome of the recombinant *P. pastoris* KM71 chromosomally harboring multiple copies of *dpgA* gene previously generated by transforming the KM71 host with pPIC3.5K_D-PhgAT. The transformants were first selected on YPD plates containing 25 μg mL^-1^ of zeocin. Subsequently, the resistant clones were re-streaked on YPD plates with higher concentration of zeocin (100, 500, 1000, 2000 and 5000 μg mL^-1^) to select for clones having multiple copies of *groEL* and *groES* genes. The copy numbers of the genes integrated were determined by quantitative real-time PCR.

### Real-time PCR

Quantitative real time-PCR was performed in duplicates using the QuantiTect SYBR Green PCR Kit (QIAGEN) in 20 μL reaction mixture containing 2 ng of genomic DNA and 0.5 μM of each primer. The thermal cycling conditions started with 15 min at 95°C followed by 40 cycles of 30 s at 95°C, 30 s at 60°C and 45 s at 72°C (Rotor-Gene RG-3000, Corbett Research). After the amplification, a melting curve analysis with a temperature gradient of 0.1°C s^-1^ from 65°C to 95°C was examined to exclude the amplification of nonspecific products. For correct determination of the starting copy quantity, the reference gene actin was quantified in parallel. The normalized copy number was calculated by relative quantification as described by Livak and Schmittgen [43] with the following formula; n = 2^-ΔΔCt^ where ΔΔCt = (Ct target sample – Ct reference sample) – (Ct target calibrator – Ct reference calibrator). Ct was defined as the point at which the fluorescence level rose above the baseline. The *P. pastoris* KM71 chromosomally harboring single copy each of the *dpgA*, *groEL*, *groES* gene from the pAO_D-PhgAT_GAP_ELS plasmid was used as the calibrator.

### Expression of D-PhgAT and GroEL-ES in *P. pastoris* KM71

Each constructed strain was grown in 200 mL of BMGY (1% yeast extract, 2% peptone, 100 mM potassium phosphate, pH 6.0, 1.34% YNB, 4 x 10^-5^% biotin and 1% glycerol) medium contained in a 1000 mL shake flask with 3 extra deep side baffles and 38 mm DeLong neck (Bellco, Part no. 2543–01000) and incubated at 30°C and 200 rpm shaking until the culture reached an OD_600nm_ of 6. Cells were harvested by centrifugation at 2000 × g for 5 min at room temperature. The cells were resuspended in 40 mL of BMMY (0.5% methanol) medium to an OD_600nm_ of 30, transferred into a 500 mL shake flask with 3 extra deep side baffles and 38 mm DeLong neck (Bellco, Part no. 2543–00500) and incubated at the desired temperature to induce the expression of the recombinant D-PhgAT and GroEL-ES. At specified time point, the culture was harvested by centrifugation. For time course study of enzyme expression, methanol was added to the culture to a final concentration of 0.5% at every 24 h in addition to that at the beginning of the induction of gene expression. For viable cell count determination, culture samples were 10-fold serially diluted with 100 mM potassium phosphate, pH 6, and 100 μL aliquots of the 10^-6^ dilutions were plated on YPD agar. The plates were incubated at 30°C for 72 h before viable cell counts were manually scored. Determinations were done in triplicates.

In the experiments with chemical additives, when the BMGY culture reached an OD_600nm_ of 6, sorbitol was added to a final concentration of 0.5 M and incubated at the desired temperature for 1 h. Cells were pelleted, resuspended in BMMY plus 0.5 M sorbitol, and the incubation was continued for the specified period before the cells were harvested and analyzed. When benzyl alcohol was used as an additive, the BMGY culture at the OD_600nm_ of 6 was added with benzyl alcohol to a final concentration of 0.4 mM and incubated at the desired temperature for 1 h. Cells were pelleted, resuspended in BMMY plus 0.4 mM benzyl alcohol, and incubated at the desired temperature with shaking for the specified period before the cells were harvested and analyzed.

### Preparation of cell-free extracts

Protein extraction was accomplished by using a French Press (FA-081A, Thermo Electron Corp.). Cell pellets were washed once and resuspended in chilled lysis buffer (50 mM sodium phosphate, pH 7.4, 1 mM PMSF, 1 mM EDTA and 5% glycerol) with the ratio of cell wet weight to buffer volume of 1:4. Each sample was subjected to 16000 psi cell pressure for 4 passes. The cell lysate was centrifuged at 12000 x g for 10 min at 4°C and the supernatant was collected for determining the D-PhgAT activity.

Protein concentration was determined using the Bradford dye-binding protein assay (Bio-Rad) with bovine serum albumin as a standard.

### Purification of the 6xHis-tagged D-PhgAT using immobilized cobalt affinity chromatography

After cultivation, cell-free extract was prepared as described above using lysis buffer without EDTA. Protein purification was performed using an AKTAprime protein chromatography system (GE Healthcare). The BD TALON^TM^ (BD Biosciences) metal affinity resin was packed in a chromatographic column (Tricorn 10/150, GE Healthcare) to a bed volume of 9 mL. The column was pre-equilibrated with 10 column volumes of binding buffer (50 mM sodium phosphate, pH 7.4, containing 10 μM PLP and 0.1 M NaCl). A 10 mL aliquot of the cell-free extract sample was applied onto the column by means of an injection loop at a flow rate of 1 mL·min^-1^ (linear flow rate = 76.4 cm·h^-1^). The column was washed with 5 column volumes of the same buffer to remove the unbound materials. The proteins were eluted with a linear gradient of 0–60 mM imidazole contained in the binding buffer for 15 column volumes at the same flow rate. Eluted proteins were collected in 3 mL fractions. Fractions containing D-PhgAT were located by D-PhgAT activity assay and the active fractions were pooled. By means of an Amicon Ultra Centrifugal Filter Device (Millipore) with a 50 kDa cut-off membrane, the pooled active fractions were concentrated and washed 3 times with 20 mL of buffer (50 mM sodium phosphate, pH 7.4, 1 mM PMSF, 1 mM EDTA and 5% glycerol). The purity of the enzyme was assessed by SDS-PAGE.

### Determination of D-phenylglycine aminotransferase activity

The D-PhgAT activity assay was performed using the published method [44] with some modifications. In a 1 mL reaction, 20 μL of soluble enzyme fraction was mixed into 980 μL of reaction mixture containing 100 mM CAPSO, pH 9.0, 10 mM D-4-hydroxyphenylglycine, 10 mM α-ketoglutaric acid, 25 μM EDTA and 25 μM PLP. The rate of 4-hydroxybenzoylformate formation was measured as a function of time by monitoring the increase in the absorbance at 340 nm using a spectrophotometer (Biospec-1601, Shimadzu Corp., Japan).

### SDS-polyacrylamide gel electrophoresis

Total cellular proteins were analyzed by preparing the samples as followed [45]. A 3 mg wet weight of cell pellet was washed once and resuspended in 0.3 mL of distilled water, then an equal volume of 0.2 M NaOH was added. After 5 min incubation at room temperature, the cells were collected and the supernatant was carefully removed. A 70 μL volume of SDS sample buffer was added to each pellet and mixed by repeatedly pipetting. The samples were boiled for 3 min to solubilize the cell contents, and centrifuged briefly. A 15 μL of cell extract in the sample buffer of each sample was subjected to SDS-polyacrylamide gel electrophoresis in 4% stacking- and 12% separating gel (Mini-Protein II Dual Slab Cell, Bio-Rad) at 150 V for 1 h and the gel was stained with Coomassie Brilliant Blue.

To distinguish between soluble and insoluble proteins, the cell lysate obtained after cell disruption by French Press was centrifuged at 12000 x g for 10 min at 4°C. The supernatant containing soluble proteins was carefully separated from the pellet containing insoluble proteins. Lysis buffer equal to the volume of the supernatant removed was added to dissolve the pellets. The protein samples from the soluble and insoluble fractions were mixed with SDS-sample buffer before boiling for 10 min, and then centrifuged briefly. For each sample, 30 microgram of protein was loaded into the gel.

## Competing interests

The authors declare that they have no competing interests.

## Authors' contributions

KJ designed and performed the experiments, analyzed data and drafted the manuscript. PL participated in planning the experiments and interpretation of results. VM criticized and gave suggestions on the results. SW conceived the study, followed up, supervised the research work and validated all data. KJ and SW wrote the paper. All authors read and approved the final manuscript.

## Supplementary Material

Additional file 1 **Genetic maps of pAO_D-PhgAT_AOX_ELS and pAO_D-PhgAT_GAP_ELS plasmids.** (A) In the pAO_D-PhgAT_AOX_ELS, the *dpgA* gene was under the control of an *AOX1* promoter, the *groEL* and *groES* were individually under the control of an *AOX1* promoter. (B) In the pAO_D-PhgAT_GAP_ELS, the *dpgA* gene was under the control of an *AOX1* promoter while the *groEL* and *groES* were individually under the control of a *GAP* promoter. Click here for file

Additional file 2 Genetic map of the pPICZ_26S rDNA_GAP_GroELS plasmid. Click here for file

Additional file 3 **DNA sequence alignment comparing the wild-type *Pseudomonas stutzeri dpgA *gene with the synthetic *dpgA *gene codon-optimized for expression in *P. pastoris.*** The codon-optimized *dpgA* gene was modified at the C-terminus with (His)_6_-Cys coding sequence before the stop codon to facilitate protein purification by immobilized ion affinity chromatography and to serve as a site specific for enzyme immobilization at the terminal Cys. Click here for file
