# Supplementary figures and images for: Effective enhancement of Pseudomonas stutzeri D-phenylglycine aminotransferase functional expression in Pichia pastoris by co-expressing Escherichia coli GroEL-GroES
Source: Microb Cell Fact. 2012 Apr 19;11:47. doi: 10.1186/1475-2859-11-47 (PMC3503884; doi:10.1186/1475-2859-11-47)

**A**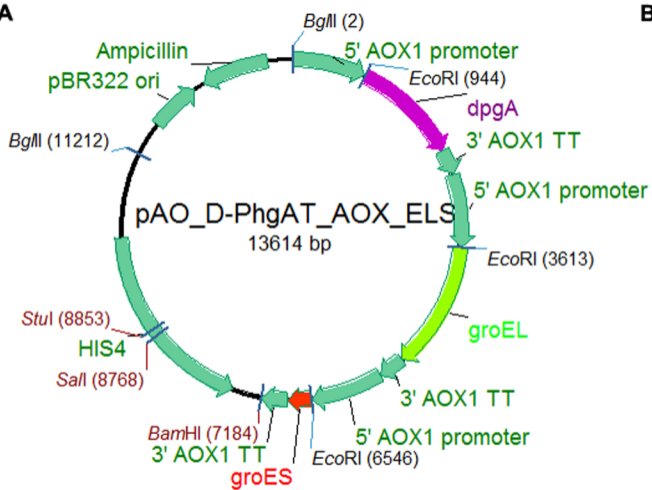**B**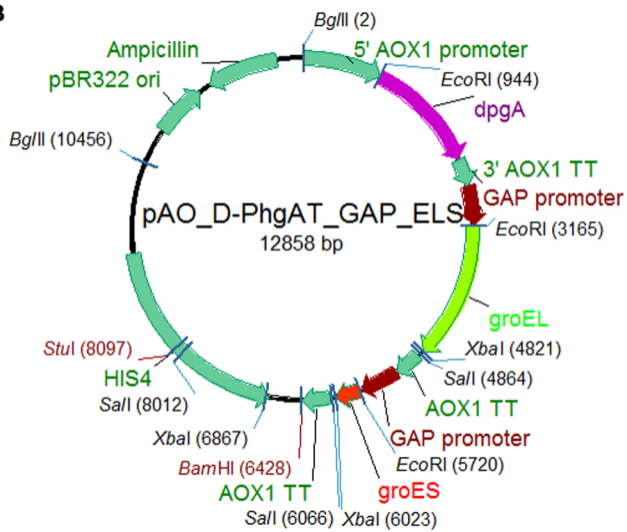

Supplement: Additional file 1 — Genetic maps of pAO_D-PhgAT_AOX_ELS and pAO_D-PhgAT_GAP_ELS plasmids. (A) In the pAO_D-PhgAT_AOX_ELS, the dpgA gene was under the control of an AOX1 promoter, the groEL and groES were individually under the control of an AOX1 promoter. (B) In the pAO_D-PhgAT_GAP_ELS, the dpgA gene was under the control of an AOX1 promoter while the groEL and groES were individually under the control of a GAP promoter. [file 1475-2859-11-47-S1.pdf]

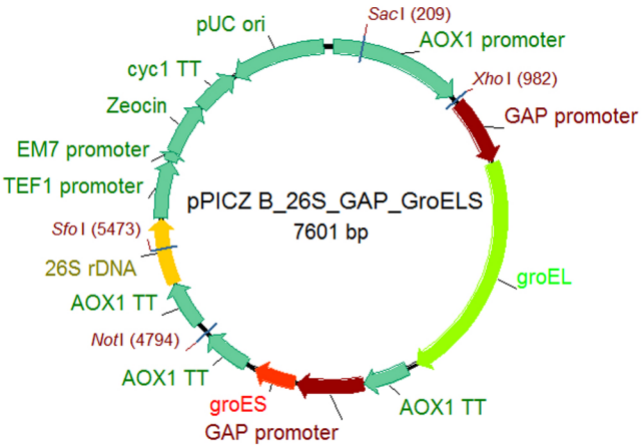

Supplement: Additional file 2 — Genetic map of the pPICZ_26S rDNA_GAP_GroELS plasmid. [file 1475-2859-11-47-S2.pdf]
