# Supplementary material for: Effective enhancement of Pseudomonas stutzeri D-phenylglycine aminotransferase functional expression in Pichia pastoris by co-expressing Escherichia coli GroEL-GroES
Source: Microb Cell Fact. 2012 Apr 19;11:47. doi: 10.1186/1475-2859-11-47 (PMC3503884; doi:10.1186/1475-2859-11-47)
Supplement: Additional file 3 — DNA sequence alignment comparing the wild-type Pseudomonas stutzeri dpgA gene with the synthetic dpgA gene codon-optimized for expression in P. pastoris. The codon-optimized dpgA gene was modified at the C-terminus with (His)6-Cys coding sequence before the stop codon to facilitate protein purification by immobilized ion affinity chromatography and to serve as a site specific for enzyme immobilization at the terminal Cys. [file 1475-2859-11-47-S3.pdf]

```
WT_dpgA    1    ATGTCGATCCTTAACGACTACAAACGTAAGACAGAAGGCTCAGTATTTTGGGCACAACGC
OPT_dpgA   1    ATGTCCATCCTGAACGACTACAAGAGAAAGACTGAGGGTTCTGTCTTCTGGGCTCAACGT
                 M  S  I  L  N  D  Y  K  R  K  T  E  G  S  V  F  W  A  Q  R

WT_dpgA    61   GCTCGGTCGGTCATGCCCGACGGCGTAACCGCAGACACCCGAGTATTTGACCCACACGGC
OPT_dpgA   61   GCTCGTTCTGTCATGCCTGATGGAGTTACTGCCGATACTAGAGTCTTCGATCCACACGGT
                 A  R  S  V  M  P  D  G  V  T  A  D  T  R  V  F  D  P  H  G

WT_dpgA    121  CTTTTCATTAGTGACGCCCAAGGCGTTCACAAGACCGATGTAGACGGCAATGTGTACCTA
OPT_dpgA   121  TTGTTCATCTCTGACGCTCAAGGAGTTCATAAGACTGACGTCGATGGTAACGTTTACTTG
                 L  F  I  S  D  A  Q  G  V  H  K  T  D  V  D  G  N  V  Y  L

WT_dpgA    181  GACTTTTTTGGCGGGCACGGAGCCCTCGTACTAGGTCATGGCCATCCTCGGGTTAACGCA
OPT_dpgA   181  GACTTCTTCGGTGGTCACGGTGCTCTGGTCTTGGGTCATGGTCATCCTCGTGTCAACGCC
                 D  F  F  G  G  H  G  A  L  V  L  G  H  G  H  P  R  V  N  A

WT_dpgA    241  GCCATCGCCGAAGCTCTTAGCCATGGCGTCCAGTACGCGGCCAGCCACCCACTGGAAGTG
OPT_dpgA   241  GCTATTGCTGAGGCTCTGTCCCACGGTGTCCAGTACGCTGCTTCTCATCCTCTGGAGGTT
                 A  I  A  E  A  L  S  H  G  V  Q  Y  A  A  S  H  P  L  E  V

WT_dpgA    301  CGATGGGCAGAACGCATCGTGGCCGCATTTCCCTCAATTCGTAAACTGCGCTTCACCGGA
OPT_dpgA   301  AGATGGGCCGAGAGAATCGTTGCTGCTTTCCCTTCCATCCGTAAGTTGAGATTCACTGGA
                 R  W  A  E  R  I  V  A  A  F  P  S  I  R  K  L  R  F  T  G

WT_dpgA    361  AGCGGCACCGAAACTACGCTGCTGGCTTTGCGGGTAGCTCGTGCCTTCACGGGCCGCCGC
OPT_dpgA   361  TCTGGTACTGAGACTACCCTGTTGGCCTTGAGAGTTGCTAGAGCTTTCACTGGTAGACGT
                 S  G  T  E  T  T  L  L  A  L  R  V  A  R  A  F  T  G  R  R

WT_dpgA    421  ATGATACTGCGCATCGCCACTCATTATCATGGCTGGCACGATTTTTCCGCATCTGGTTAT
OPT_dpgA   421  ATGATTTTGAGAATCGCTACTCATTACCACGGTTGGCATGATTTCTCCGCCTCTGGTTAC
                 M  I  L  R  I  A  T  H  Y  H  G  W  H  D  F  S  A  S  G  Y

WT_dpgA    481  AACAGCCATTTCGATGGCCAGCCGGCGCCGGGCGTGCTACCTGAAATTGCGAAGAATACT
OPT_dpgA   481  AACTCCCACTTCGACGGTCAGCCTGCTCCAGGTGTCTTGCCAGAAATCGCCAAGAACACT
                 N  S  H  F  D  G  Q  P  A  P  G  V  L  P  E  I  A  K  N  T

WT_dpgA    541  TTGCTGATTCGCCCTGATGATATTGAAGGCATGCGAGAAGTTTTCGCGCAGCATGGCAGC
OPT_dpgA   541  CTGTTGATTCGTCCTGACGACATCGAGGGTATGAGAGAGGTTTTCGCTCAACATGGATCT
                 L  L  I  R  P  D  D  I  E  G  M  R  E  V  F  A  Q  H  G  S

WT_dpgA    601  GACATTGCAGCATTCATTGCCGAACCTGTGGGTTCGCACTTTGGCGTCACTCCAGTGAGC
OPT_dpgA   601  GACATCGCTGCCTTCATCGCTGAACCAGTTGGATCTCACTTCGGTGTTACTCCAGTCTCT
                 D  I  A  A  F  I  A  E  P  V  G  S  H  F  G  V  T  P  V  S

WT_dpgA    661  GATAGCTTTCTACGCGAAGGCGCAGAATTGGCTCGGCAATACGGTGCCCTGTTCATCCTA
OPT_dpgA   661  GACTCCTTCTTGAGAGAGGGTGCTGAATTGGCTAGACAGTACGGTGCTCTGTTCATCCTT
                 D  S  F  L  R  E  G  A  E  L  A  R  Q  Y  G  A  L  F  I  L

WT_dpgA    721  GACGAAGTAATTTCTGGTTTCCGGGTCGGGAATCACGGAATGCAGGCGCTCCTTGATGTT
OPT_dpgA   721  GACGAGGTTATCTCCGGATTCCGTGTCGGTAACCATGGTATGCAGGCTTTGCTGGACGTT
                 D  E  V  I  S  G  F  R  V  G  N  H  G  M  Q  A  L  L  D  V

WT_dpgA    781  CAGCCGGATCTCACCTGCCTGGCTAAGGCCAGCGCAGGCGGGCTTCCCGGTGGCATCTTG
OPT_dpgA   781  CAGCCAGATTTGACTTGTTTGGCTAAAGCCTCTGCCGGAGGACTTCCAGGAGGTATCTTG
                 Q  P  D  L  T  C  L  A  K  A  S  A  G  G  L  P  G  G  I  L
```

```
WT_dpgA    841  GGCGGGCGCGAAGATGTCATGGGAGTTCTCAGCCGAGGCAGTGATCGCAAGGTACTACAT
OPT_dpgA   841  GGAGGTAGAGAAGATGTCATGGGAGTCTTGTCCCGTGGTTCCGACCGTAAAGTCTTGCAT
                 G  G  R  E  D  V  M  G  V  L  S  R  G  S  D  R  K  V  L  H

WT_dpgA    901  CAGGGTACTTTTACCGGCAACCCGATTACTGCGGCAGCGGCGATCGCAGCCATCGACACC
OPT_dpgA   901  CAAGGTACTTTCACCGGAAATCCTATCACCGCCGCTGCTGCCATCGCTGCTATTGACACC
                 Q  G  T  F  T  G  N  P  I  T  A  A  A  A  I  A  A  I  D  T

WT_dpgA    961  ATCCTTGAAGACGATGTTTGCGCGAAGATCAATGACCTTGGTCAATTCGCCAGGGAGGCG
OPT_dpgA   961  ATCTTGGAGGATGACGTTTGTGCTAAGATTAACGACTTGGGTCAATTCGCCAGAGAAGCT
                 I  L  E  D  D  V  C  A  K  I  N  D  L  G  Q  F  A  R  E  A

WT_dpgA    1021 ATGAATCATCTATTTGCCCGCAAGGGACTGAACTGGCTGGCCTATGGTCGCTTCTCAGGC
OPT_dpgA   1021 ATGAATCACTTGTTCGCTAGAAAGGGTCTGAACTGGTTGGCTTACGGTAGATTCTCCGGA
                 M  N  H  L  F  A  R  K  G  L  N  W  L  A  Y  G  R  F  S  G

WT_dpgA    1081 TTCCACCTGATGCCGGGGCTGCCACCTAATACAACCGACACCGGCTCCATAACCCGAGCT
OPT_dpgA   1081 TTCCATCTGATGCCTGGTTTGCCACCTAACACCACTGATACCGGTTCCATCACTCGTGCC
                 F  H  L  M  P  G  L  P  P  N  T  T  D  T  G  S  I  T  R  A

WT_dpgA    1141 GAAGTCGCACGCCCCGATGTGAAGATGATCGCAGCAATGCGCATGGCATTGATATTGGAA
OPT_dpgA   1141 GAAGTCGCTAGACCTGACGTTAAGATGATCGCCGCTATGAGAATGGCCTTGATCCTGGAG
                 E  V  A  R  P  D  V  K  M  I  A  A  M  R  M  A  L  I  L  E

WT_dpgA    1201 GGTGTGGATATCGGCGGGCGCGGGTCAGTTTTCCTGTCAGCACAGCATGAACGCGAACAT
OPT_dpgA   1201 GGTGTTGACATTGGTGGTAGAGGTTCCGTCTTCCTGTCCGCCCAACACGAACGTGAGCAT
                 G  V  D  I  G  G  R  G  S  V  F  L  S  A  Q  H  E  R  E  H

WT_dpgA    1261 GTTGAGCATCTGGTGACAACCTTTGATCGCGTATTAGACCGCCTGGCGGACGAAAACCTG
OPT_dpgA   1261 GTCGAACACCTTGTCACCACTTTCGATAGAGTTCTGGATCGTTTGGCCGATGAGAACCTG
                 V  E  H  L  V  T  T  F  D  R  V  L  D  R  L  A  D  E  N  L

WT_dpgA    1321 TTGTCTTGGCAACCAACTAATTTGTCTGGAAACCAATCATGA------------------
OPT_dpgA   1321 CTGTCCTGGCAACCAACTAACTTGTCCGGTAACCAATCCCATCACCATCACCATCACTGC
                 L  S  W  Q  P  T  N  L  S  G  N  Q  S  H  H  H  H  H  H  C

WT_dpgA    1362 ---
OPT_dpgA   1381 TAA
                 *
```
